# Regenerative repair of Pifithrin-α in cerebral ischemia via VEGF dependent manner

**DOI:** 10.1038/srep26295

**Published:** 2016-05-23

**Authors:** Ping Zhang, Xuhui Lei, Ying Sun, Haitao Zhang, Liang Chang, Chenlong Li, Daming Liu, Nishant Bhatta, Zhiren Zhang, Chuanlu Jiang

**Affiliations:** 1 Department of Neurosurgery, The Second Affiliated Hospital of Harbin Medical University, Harbin, China; 2 Department of Cardiology and Clinical Pharmacy, Harbin Medical University Cancer Hospital, Institute of Metabolic Disease, Heilongjiang Academy of Medical Science, Key Laboratories of Education Ministry for Myocardial Ischemia Mechanism and Treatment, Harbin, China

## Abstract

Promoting regenerative repair, including neurogenesis and angiogenesis, may provide a new therapeutic strategy for treatment of stroke. P53, a well-documented transcription factor, has been reported to be involved in cerebral ischemia and also serves as an important regulator of vascular endothelial growth factor (VEGF). However, the role of p53 in endogenous regenerative repair after brain ischemia is poorly understood. In this study, we investigated the effects of PFT-α, a specific p53 inhibitor on neurogenesis and angiogenesis improvement and associated signal pathways in rats impaired by cerebral artery occlusion (MCAo). PFT-α induced neuroprotection, reduced infarct volume and neurological functional impairment after ischemic stroke. More importantly, neurogenesis and angiogenesis were greatly enhanced by PFT-α, and accompanied by increased expression of VEGF. Moreover, we got consistent results in neural stem cells (NSCs) isolated from fetal rats. In contrast, application of the anti-VEGF neutralizing antibody (RB-222) partially reversed PFT-α-induced neuroprotection and rescued p53 expression. Noteworthily, inhibition of p53 after ischemic stroke in these rats improved their outcomes via promotion of regenerative repair. In conclusion, PFT-α could serve as a promising therapeutic strategy for ischemic stroke by promoting regenerative repair.

Ischemic stroke is one of the leading cause of death and disability world-wide. It not only induces cell death and ischemic penumbra, but neuronal repair itself as well^1^,^2^, eventually restoring some brain functions via neurogenesis^3^ and angiogenesis^4^. However, the degree of repairation and regeneration is insufficient to recover from brain ischemic injury and alter the course of disability or death resulting from cerebral ischemia. Therefore, identifying process that promoting regenerative repair, including neurogenesis and angiogenesis, hold great promise as a therapeutic strategy for the treatment of stroke.

P53, as a transcription factor, is critical for activation or suppression of multiple genes^5^. In addition to its established effects on tumor^6^, metabolism^7^ and cell cycle^8^, p53 has also been reported to be up-regulated in cerebral ischemia^9^,^10^. The induced p53 potentiated impairment resulting from cerebral ischemia^11^,^12^. Accordingly, Pifithrin-alpha (PFT-α), a pharmacologically developed p53 inhibitor, has gained increasing interest in recent years as a treatment for the cerebral ischemia. PFT-α has been shown to induce neuroprotection against cerebral ischemia in rats with delayed treatment^13^. Recently, we have demonstrated that PFT-α was effective in promoting the survival of grafted neural stem cells (NSCs) and improved recovery after cerebral ischemia in rats^14^. Interestingly, findings from recent reports have indicated that p53, which usually plays a critical role in sensing genotoxic and other stresses, is also an important regulator of vascular endothelial growth factor (VEGF)^15^, and VEGF has been shown that has the protective effects on PC12 cells^16^ and enhances angiogenesis in the ischemic brain^17^. However, whether VEGF as a critical target of p53 involved in PFT-α-induced neuroprotection in cerebral ischemic rats remains unknown.

Based upon these results, we hypothesized that p53 may act as a regulator of neurogenesis and angiogenesis in cerebral ischemia through VEGF signaling pathway. To test this hypothesis, the effects of PFT-α in a rat model of transient focal cerebral ischemia was utilized as a means to examine the possible associated mechanism of regenerative repair induced by PFT-α. Results demonstrated that PFT-α could improved ischemic stroke outcome by inducing neurogenesis and angiogenesis through the upregulation of VEGF expression.

## Results

### PFT-α promoted endogenous NSCs proliferation *in vivo* and *in vitro*

**Figure 1:**
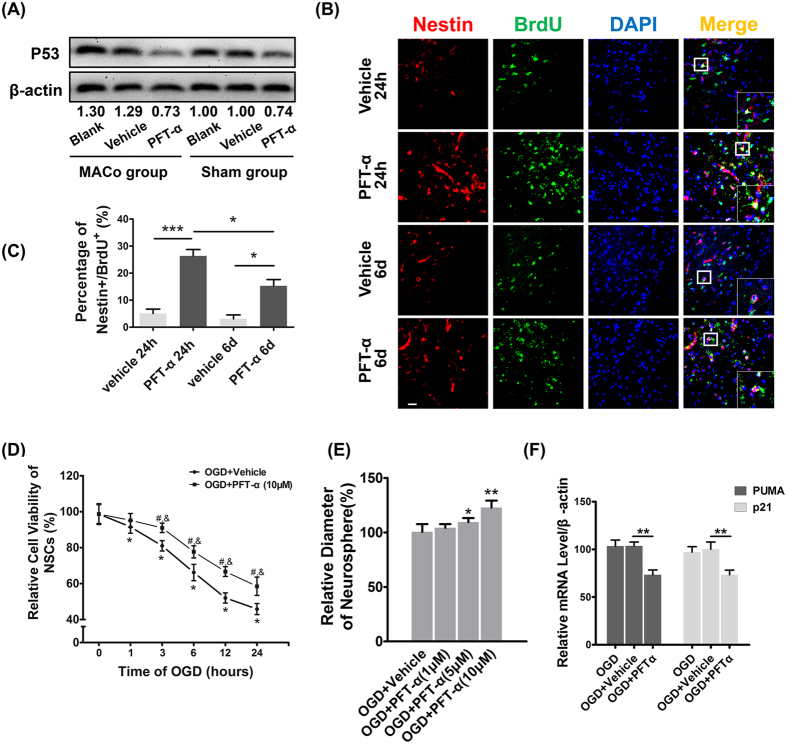
Effects of PFT-α on proliferation of NSCs *in vivo* and *in vitro*. (**A**) Protein abundance of p53 using western blot analysis of brain homogenates. β-actin served as a loading control (n = 3). (**B**) Representative photomicrographs of cells double-labeled immunofluorescence for BrdU (green) and Nestin (red) in the SVZ. Scale bar = 10 μm. (**C**) Quantitive determination of BrdU/Nestin positive cells for each group. *P < 0.05, ***P < 0.0001. Values are mean ± SEM (n = 6). (**D**) The viability of NSCs was determined by cell counting kit-8 (CCK-8) at 0–24 h after OGD (n = 3, *P < 0.05 or ^*#*^*P* < 0.05 vs. control, ^&^*P* < 0.05 vs. OGD + Vehicle, respectively). Values are mean ± SEM. (**E**) The relative diameter of Neurosphere was examed after OGD + Vehicle and OGD + PFT-a (1 μM, 5 μM, 10 μM) (n = 6, *P < 0.05 or **P < 0.01 vs. OGD + Vehicle). Error bars show SEM. (**F**) Relative mRNA leves of PUMA and p21 was determined by qRT-PCR after OGD (n = 3, **P < 0.01). Values are mean ± SEM.

Cerebral ischemia activated endogenous NSCs proliferation in the subventricular zone (SVZ) and pathological process, such as formation of ischemic penumbra. As p53 is essential in NSCs functions^13^,^18^, we first investigated the role of p53 in proliferation of NSCs on ischemic stroke. To accomplish this goal, rats were treated with either vehicle or PFT-α at 24 h after stroke or sham. The effects of these treatments were then examined at 7 days after stroke. Results of Western Blotting showed that p53 expression was upregulated in cerebral ischemic rats and PFT-α decreased the expression of p53 compared to MCAo and Vehicle groups (Fig. 1A). Next, proliferation of endogenous NSCs was detected using BrdU/Nestin immunofluorescence at 14 days after stroke. Compared with the Vehicle group, rats treated with PFT-α showed significantly increased BrdU/Nestin-positive NSCs in the SVZ (Fig. 1B,C). These findings are consistent with the studies of Luo, *et al*. in rats with delayed treatment (6 day) of p53 inhibitor^13^. However, our results indicated that the treatment with PFT-α at earlier stage (24 h) lead to more proliferating NSCs (Fig. 1B,C), which will benefit regenerative repairation after stroke.

Next, in order to thoroughly prove the neuroprotective effects of PFT-α, we calculated the NSCs viability and neurospheres diameter *in vitro*. Consistently, administration of PFT-α will remarkably enhance proliferation of NSCs (Fig. 1D,E). Moreover, treatment with PFT-α could suppress expression of p53 target genes PUMA and p21, which further showed the p53 inhibition by PFT-α treatment (Fig. 1F).

### PFT-α stimulated neurogenesis and angiogenesis in ischemic zone

**Figure 2:**
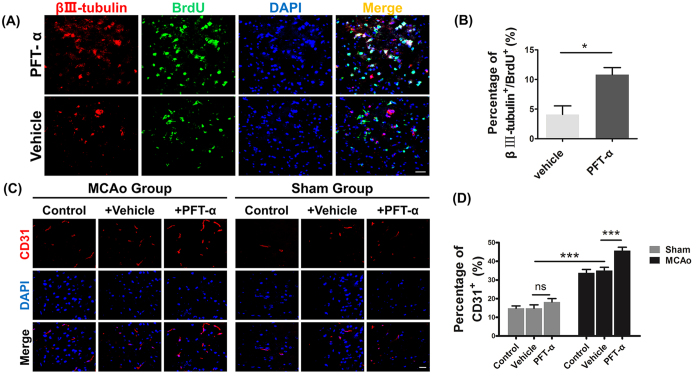
PFT-α enhanced neurogenesis and angiogenesis after transient middle cerebral artery occlusion (tMCAo) in adult rats. (**A**) Representative images of cells double-labeled for BrdU (green) and βIII-tubulin (red) in the boundary zone of ischemic core from Vehicle and PFT-α rats at 7 days after stroke. Scale bar = 10 μm. (**B**) Quantitive determination of BrdU and βIII-tubulin in double-labeled cells for each group. *P < 0.05. Values are mean ± SEM (n = 6). (**C**) Photomicrographs showed CD31 (red) and DAPI (blue) immunofluorescence staining of microvessels in the peri-infarct area from Control, Vehicle and PFT-α groups after 1 week of tMCAo or sham. Scale bars = 5 μm. (**D**) The number of microvessels was quantified (n = 6, ***P < 0.0001). Error bars show SEM.

Promotion of neurogenesis and angiogenesis after stroke is critical for neuronal self-repair. Hence, NSCs differentiation in the boundary zone and angiogenesis in ischemic area were detected using double label immunostaining. BrdU/βIII-tubulin double-positive cells were identified as new neurons^19^,^20^. In contrast to vehicle group, PFT-α significantly increased BrdU/βIII-tubulin double-positive cells in the boundary zone of impaired cortex (Fig. 2A,B). Moreover, PFT-α-treated rats presented more CD-31-positive cells, a marker for newly formed microvessels, than that obtained in rats treated with vehicle 7 days after MCAo (Fig. 2C,D). However, PFT-α treatment did not show an obvious enhancement of neo-vascularization in sham-operated rats (Fig. 2C,D). Taken together, our analysis showed that suppressing p53 activity promoted regeneration of neuronal cells.

### PFT-α reduced lesion size after stroke

**Figure 3:**
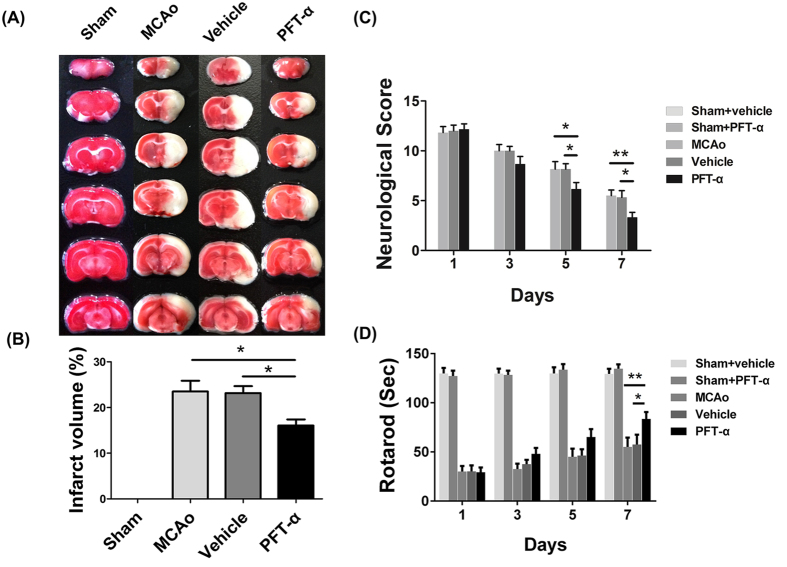
PFT-α reduced infarct volume and neurological functional impairment after stroke in rats. (**A**) Representative sets of TTC-stained brain slices (Sham group; MCAo group; Vehicle group; PFT-α group) after 7 days of transient middle cerebral artery occlusion (tMCAo). (**B**) Treatment with PFT-α significantly decreased infarct volume in cerebral ischemic rats. *P < 0.05. Data are expressed as mean ± SEM (n = 6). Functional recovery was evaluated using mNSS (**C**) and Rotarod test (**D**). The behavioral tests were performed at 1, 3 and 7 days after tMCAo. Data are mean ± SEM (n = 6). *P < 0.05, **P < 0.01.

Lesion volume caused by 90-min MCAo and following reperfusion for 7 days was determined based on quantification of TTC staining of brain slices. In sham-operated rats, no infarct volume was observed. While a significant infarct volume was observed in the MCAo and Vehicle group, the size of this lesion failed to differ significantly between these two groups. Notably, the lesion volume was significantly decreased in the PFT-α group, compared with both the MCAo and Vehicle group (Fig. 3A,B).

### PFT-α improved the behavioral recovery of MCAo rats

Neurological functions of all animals were normal before MCAo. After MCAo, 11 rats died and data from 1 rat with 0 points, 2 rats with 1 points, 3 rats with 4 points according to Longa’s scoring system were excluded from the analyses. All rats in the experiment exhibited similar levels of functional deficits at day 1 after stroke and no statistically significance existed among the groups 3-days post-stroke. However, at day 5, rats treated with PFT-α had lower scores of mNSS compared with the Vehicle and MCAo groups (Fig. 3C); but no statistically significant differences were obtained on the rotarod test (Fig. 3D). At day 7, the effects of PFT-α were statistically significant for both rotarod tests and mNSS scores (Fig. 3C,D). Statistical significance was not achieved between MCAo and Vehicle group all the time. These results demonstrated that PFT-α treatment improved the functional behavioral outcomes in rats after stroke.

### PFT-α induced VEGF expression *in vivo* and *vitro*

**Figure 4:**
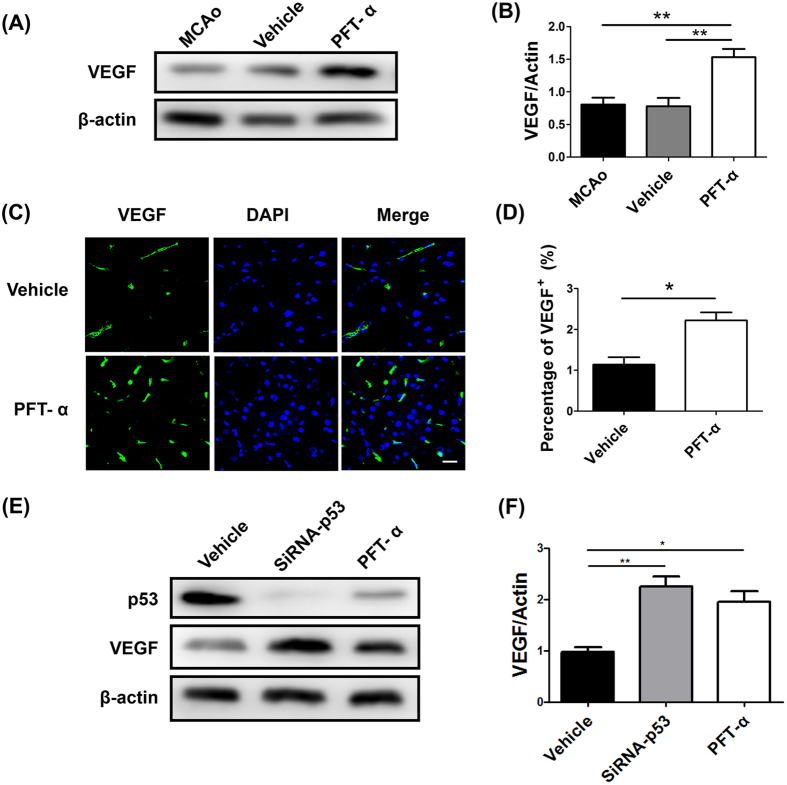
Increased expression of VEGF after inhibition of p53 *in vivo* and *vitro*. (**A**) Protein abundance of VEGF 7 days post-stroke using western blot analysis of brain homogenates (MCAo group; Vehicle group; PFT-α group). β-actin served as a loading control. (**B**) Quantitative determination showed that PFT-α significantly increased the expression of VEGF in cerebral ischemic rats. **P < 0.01. Values are mean ± SEM (n = 3). (**C**) Representative photomicrographs of VEGF (green) and DAPI (blue) immunostaining from Vehicle and PFT-α rats at 7 days after stroke. Scale bar = 5 μm. (**D**) Quantitative determination of VEGF-positive area for each group. Values are mean ± SEM (n = 6). *P < 0.05. (**E**) Protein abundance of VEGF in NSCs using western blot analysis (Vehicle group; siRNA-p53 group; PFT-α group). (**F**) Quantitative determination showed that siRNA-p53 or PFT-α significantly increased the expression of VEGF in NSCs. **P < 0.01, *P < 0.05. Values are mean ± SEM (n = 3).

As previous researches revealed, VEGF is necessary for cell-induced functional recovery and vascular repair, and its expression could be affected by p53 transcriptional regulatory^21^,^22^. Thus, to determine whether VEGF was upregulated in response to PFT-α treatment after stroke, we performed an immunofluorescent analysis at 7 days after cerebral ischemia. Results showed that the expression of VEGF in PFT-α-treated rats was significantly higher than that in vehicle-treated rats (Fig. 4C,D), and the results were confirmed with Western Blot assays (Fig. 4A,B). To further identify that the up-regulation of VEGF was induced by p53 inhibition, neural stem cells from SVZ were extracted and processed with vehicle, p53-siRNA or PFT-α. Analysis of protein expression indicated that VEGF was significantly elevated as a consequence of p53 knockdown, which was consistent with PFT-α treatment *in vivo* (Fig. 4E,F).

### Inhibition of VEGF exacerbated stroke

**Figure 5:**
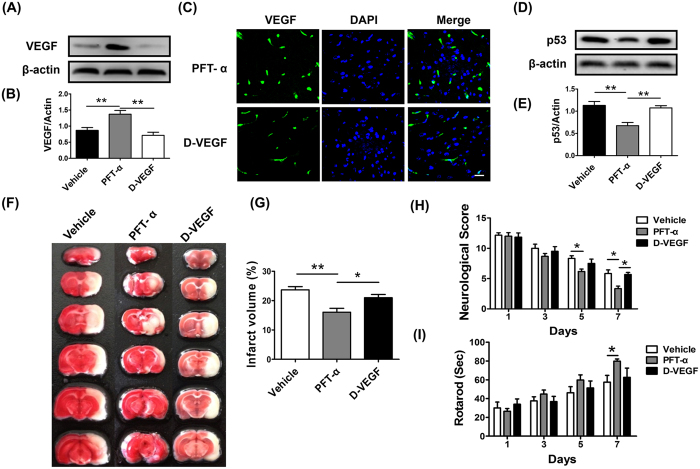
Inhibition of VEGF partly abrogated the neuroprotection of PFT-α in cerebral ischemic rats. (**A**) Protein abundance of VEGF post-stroke using western blot analysis of brain homogenates (Vehicle group; PFT-α group; D-VEGF group). β-actin served as a loading control. (**B**) Quantitative determination showed that RB-222 significantly decreased the expression of VEGF in PFT-α-treated rats. **P < 0.01(n = 3). (**C**) Representative photomicrographs of VEGF (green) and DAPI (blue) immunostaining from PFT-α and D-VEGF rats at 7 days after stroke. Scale bar = 5 μm. (**D**) Protein abundance of p53 post-stroke using western blot analysis of brain homogenates. β-actin served as a loading control. (**E**) Quantitative determination showed that RB-222 significantly increased the expression of p53 in PFT-α-treated rats. **P < 0.01(n = 3). (**F**) Representative sets of TTC-stained brain slices after 7 days of tMCAo. (**G**) Treatment with RB-222 increased infarct volume in PFT-α-treated rats. *P < 0.05 (n = 6). Functional recovery was evaluated using mNSS (**H**) and Rotarod test (**I**). The behavioral tests were performed at 1, 3 and 7 days after tMCAo. *P < 0.05 (n = 6). Data are expressed as mean ± SEM.

Given the degree that VEGF was elevated after the administration of PFT-α, we next investigated importance of VEGF in PFT-α-induced neuroprotection after cerebral ischemia. Neutralizing antibody against VEGF (RB-222, NeoMarkers), which effectively inhibits the expression of VEGF, was used to accomplish this goal (D-VEGF group). The results showed that RB-222 decreased VEGF expression compared to PFT-α group in cerebral ischemic rats (Fig. 5A,B), and the results were confirmed by immunofluorescent analysis either (Fig. 5C). Intriguingly, the expression of p53 in the D-VEGF group was increased compared to that in the PFT-α group (Fig. 5D,E).

To investigate whether neuroprotection of PFT-α was blocked following VEGF neutralization, infarct volumes and neurological functional deficits were detected in different groups. We observed that inhibition of VEGF potentiated infarct volume compared to PFT-α group (Fig. 5F,G). At 1, 3, 5 days after MCAo, all groups treated with RB-222 presented no effect based upon results obtained with rotarod and mNSS tests. However, at 7days post-MCAo, rats treated with RB-222 had greater mNSS scores compared with that in PFT-α group (Fig. 5H), but no therapeutic effect on rotarod test was observed (Fig. 5I). The results suggested that inhibition of VEGF may partially abrogate the improvement of infarct volume and behavioral functions induced by PFT-α.

## Discussion

Neurogenesis^1^,^23^,^24^,^25^,^26^ and angiogenesis^27^, as associated with stroke, can contribute to brain self-repair, thereby improving the outcome of this condition. In present study, we examined the effects of PFT-α as a treatment strategy for promoting regenerative repair and explored some of its latent mechanisms in a cerebral ischemic rat model. Administration of PFT-α, a p53 inhibitor, starting from the first day after tMCAo, benefited both neurogenesis and angiogenesis, thereby improving stroke outcome.

P53 is an important mediator in cerebral ischemic injury and induces cell death after cerebral ischemia^11^,^28^,^29^. Meanwhile, ischemic stroke could activate the expression of p53^30^,^31^. Further evidence supporting a damaging role for this transcription factor that attenuation of p53 expression protected against focal ischemic injury in p53 knockout mice^32^ and the p53 inhibitor, PFT-α, reduced focal damage induced by ischemic stroke^29^,^33^. In our previous study, PFT-α was used to pretreat NSCs, which were then transplanted to the rats after stroke. We found that the treatment increased the survival rate of grafted NSCs and improved functional recovery in the stroke model^14^. However, logistic and ethical concerns may limit the clinical application of this treatment. In the current study, rats were treated with PFT-α intracerebroventricularly at 24 h after tMCAo, a temporal window when most of stroke patients could be treated. We observed that delivering PFT-α not only promoted neurogenesis and angiogenesis, but also reduced infarct volume and enhanced behavioral outcomes at 7 days after MCAo in rats. Thus, induction of NSCs endogenous regeneration will solid the scientific foundation for expanding PFT-α application in clinical stroke management.

De novo neurogenesis was found in the SVZ of adult mammalian brain after stroke^34^. However, most of these newly developed cells die within weeks after the ischemic injury and the ability to recover from behavioral deficits in these stroke animals are deficient^23^. A major basis for this deficiency is that impaired brain tissue does not provide an adequate environment for these new cells^35^. Interestingly, it has been reported that loss of p53 function leads to elevated neurogenesis in the developing telencephalon *in vivo* and in cultured NPCs^36^. Accordingly, we reasoned that PFT-α might promote neurogenesis after stroke. In fact, we did observe an increase of BrdU/βIII-tubulin positive staining in the boundary zone of ischemic core.

Angiogenesis has been shown to be an essential part of neurorestorative events after stroke. To the best of our knowledge, the role of p53 in angiogenesis after cerebral ischemia had not been investigated. It has been shown that PFT-α may be a novel therapeutic strategy for improving angiogenetic disorders induced by AngII in the heart^37^. Therefore, we speculated that PFT-α may have similar effects in cerebral ischemia. In the present study, we used CD31 as markers for microvessels and demonstrated that repairs after cerebral ischemia by PFT-α treatment appear to involve angiogenesis. Fan, *et al*. reported that VEGF is associated with neovascularization in the hypoxic-ischemic brain^38^. Furthermore, PFT-α blockade p53 nucleus and mitochondria location^28^ and abrogated the decrease of VEGF secretion from cardiac microvascular endothelial cells induced by AngII^37^. We have previously observed that PFT-α inhibited p53 function *in vivo* by blockade of the translocation of p53 to the nucleus, either^14^. Based upon these results, we demonstrated that PFT-α increased the expression of VEGF, a protein which is upregulated^39^ and leads to angiogenesis^15^ in cerebral ischemia and neurogenesis in depressive disorders^40^. Since upregulation of VEGF promoted angiogenesis and neurogenesis, which further improved physiological functions after cerebral ischemic injury^41^, it is reasonable to propose that PFT-α promotes neurogenesis and angiogenesis, at least in part, through the upregulation of VEGF expression. To examine this possibility, we used a neutralizing antibody against VEGF (RB-222, NeoMarkers) as a means of inhibiting the expression of VEGF. When evaluated at 7 days after stroke, inhibition of VEGF was partly abrogated followed by improvements in infarct volume and behavioral functions in PFT-α treatment. Further, the reduced-p53 protein expression was significantly restore after VEGF-neutralization, which is consistent with former reported in hepatocellular carninoma^42^. One possibility is that activation of PI3K/AKT signaling pathway stimulated by VEGF stabilized and phosphorylated MDM2, leading to the ubiquitination of P53^43^,^44^,^45^. However, the latent mechanism is complicated and deserved further investigation. Taking together, these results substantiated our hypothesis that PFT-α promoted regenerative repair, including neurogenesis and angiogenesis, at least in part, through the upregulation of VEGF expression.

In conclusion, our studies showed an effect of PFT-α in promoting neurogenesis and angiogenesis through the VEGF signal pathway in a tMCAo model, suggesting a new potential mechanism of PFT-α for stroke treatment, highlighting a key target of p53 as a mediator of brain repair for a disorder frequently difficult to treat after its occurrence. This work serves as a foundation for future studies aimed at providing a more comprehensive understanding regarding the signaling pathway of critical p53 protein in cerebral ischemia.

## Materials and Methods

### Animals

Adult male Sprague-Dawley (SD) rats were used in the experiments. The rats were kept at room temperature with a 12 h light/dark cycle following surgery. They had access to food and water ad libitum and were food-deprived for 12 h before surgery. The study was approved by the ethics committee of Harbin Medical University and conducted according to the National Institutes of Health (NIH) Guide. All efforts were made to minimize animal suffering during the experiments.

### Induction of the focal cerebral ischemia

Rats weighing 200–230 g were subjected to transient middle cerebral artery occlusion (tMCAo) according to procedures described previously with minor modification^46^. In brief, rats were anesthetized with an intraperitoneal injection of 10% chloral hydrate (350 mg/kg). The right common carotid artery (CCA) was exposed after a midline skin incision. A nylon suture (0.24 mm diameter) with a rounded tip was inserted from the right external carotid artery (ECA) into the internal carotid artery (ICA) and occluded the middle cerebral artery. The suture was withdrawn at 90 min after MCAo. Sham group was performed by using the same procedure without suture insertion. The body temperature was maintained at 37 °C using a homeothermic blanket during surgery.

### Drug administration

For the PFT-α group, 3 μl of PFT-α (10 μM) were injected intracerebrally 24 h after tMCAo for 3 consecutive days^14^,^47^. Vehicle-treated animals received 3 μl of DMSO (2%) instead. For the D-VEGF group, to achieve inhibition of VEGF on basis of PFT-α treatment, additional injections for intracerebral delivery of an anti-VEGF neutralizing antibody (10 μg of RB-222 (NeoMarkers) were diluted in PBS to a final volume of 10 μl) were performed 1 Day before MCAo using the same protocol as for PFT-α injection. Following treatments, the incisions were sutured and the animals was returned to their cages for recovery from the anesthesia. Treatment of rats was assigned in a blinded and randomized manner.

### BrdU labeling

5-Bromo-2-deoxyuridine (BrdU, Sigma) is the thymidine analog used for mitotic labeling. To investigate cell proliferation and differentiation, BrdU (50 mg/kg × 2, i.p.) was administered beginning on Day 4 after stroke or sham surgery for 3 consecutive days (N = 6 rats per group). The rats were euthanized 1 day after the final BrdU administration.

### Immunofluorescence and cell counting

All procedures for immunofluorescence were performed as previously reported^48^. Frozen brain tissues were cut into 20 μm-thick coronal slices. Sections were immunostained with 10% BSA in PBS, and incubated with the primary antibodies BrdU (1:200; Sigma), VEGF (1:25; Abcam) or CD31 (1:50; Santa Cruz Biotechnology) overnight at 4 °C. Some sections were double incubated with BrdU/Nestin (1:200; Sigma) or BrdU/βIII-tubulin (1:200; Cell Signaling Technology). Secondary antibodies were Alexa Fluor 594 and 488-labeled IgG (1:1000; Invitrogen). Nuclei were counterstained with DAPI. For BrdU immunostaining, all sections were incubated in 2 N HCl at 37 °C for 30 min before blocking to denature DNA as described previously^49^. Images were obtained using an Olympus FV 1,000 confocal microscope. For cell counting, two sections from each animal were randomly selected and the number of positive cells were counted at 3 random fields per section^50^. Positive cells were measured using Image-Pro Plus version 6.0 (Media Cybernetics, USA) software.

### Western Blotting

Briefly, fresh brain tissue was lysed with lysis buffer. The protein concentration was determined using BCA protein assay. Equal amounts of total proteins samples were subjected to SDS-PAGE and blotted onto PVDF membranes. PVDF membranes were blocked with 5% skim milk in TBST buffer and then incubated with primary antibodies against p53 (1:500; Abcam) and VEGF (1:1000; Abcam) at 4 °C overnight. Subsequently, the corresponding horseradish peroxidase-conjugated secondary antibody was applied. The results were visualized with an imaging system and quantified by scanning densitometry using the Quantity One Software (Bio-Rad, Hercules, CA, USA). Three independent detections were performed.

### TTC staining and measurement of infarct volume

Seven days after sham or MCAo surgery, the rats were decapitated under deep anesthesia. Their brains were carefully removed and sectioned into six 2.0 mm thick coronal sections. Sections were stained with 2% TTC in normal saline for 30 min and then fixed in 4% paraformaldehyde solution overnight. Digital images of six adjoining sections from each animal were taken. The size of infarct regions was determined by subtracting the size of the ipsilateral hemisphere from that of the contralateral hemisphere plus the size of non-TTC staining. Infarct volume was calculated by summing of the infarct size of six sections multiplied by section thickness^51^,^52^. All infarct measurements were performed by an individual who was blind to the treatment.

### Neurological function tests

Two investigators who were blinded to the treatment groups evaluated neurological function of all animals. After MCAo, neurological function was assessed according to Longa’s scoring system^53^. Rats scoring 0, 1, 4 points were excluded from the experiment. Rotarod motor tests^54^,^55^ and Modified Neurological Severity Scores (mNSS)^14^,^56^,^57^ were performed to evaluate neurological function 1 day before and 1, 3, 7 days after MCAo. The mNSS included tests of motor, sensory, reflex and balance performance with scores ranging from 0 (normal) to 18 (maximal deficit).

### Isolation and culture of NSCs

Neural stem cells were isolated from cortex and ganglionic eminence of 14-day-old SD fetal rats as described previously^19^,^58^. In brief, brain tissue was dissected in PBS containing streptomycin (50 μg/ml; Invitrogen) and penicillin (50 U/ml; Invitrogen). After dissection, brain tissue was triturated to a single-cell suspension using a transfer pipette. The cells were cultured in a 1:1 mixture of DMEM/F12 (Invitrogen) supplemented with 20 ng/ml b-FGF (Pepro Tech), 20 ng/ml EGF (Pepro Tech), 25 mM L-glutamine, 2% B27 (Invitrogen), 100 U/ml penicillin and 100 μg/ml streptomycin at 37 °C, 5% CO_2_, 95% humidified atmosphere. Half of the medium was replaced by equal volume of fresh medium every 3–4 days and cells were passaged every 7–9 days.

### Oxygen Glucose Deprivation and PFT-a Treatment of NSCs

To induce oxygen glucose deprivation (OGD), the NSCs (10^4^/well) were seeded in 96-well plates in 200 μL of glucose-free NBM-B27medium (Neurobasal glucose-free; Invitrogen) and incubated at 37 °C, 5% CO_2_, and 94% N_2_ in an incubator under hypoxic conditions (Thermo Scientific)^14^. A concentration of 10 μM PFT-α was added into the glucose-free NBM-B27 medium under OGD conditions.

### Cell Viability Analysis

The cell viability of NSCs cultured in OGD + Vehicle and OGD + PFT-α (10 μM) condition was evaluated at the time points of 0, 1, 3, 6, 12 and 24 h. Briefly, 20 μL of CCK-8 (Beyotime, Shanghai, China) solution was added to each well, and the cells were incubated at 37 °C for 2 h. Subsequently, the absorbance of samples was measured on a microplate reader at a wave length of 450 nm^14^. The cell growth was expressed as relative cell viability (%). Each experiment was performed in triplicate.

### Diameter Measurement of Neurospheres

Under an inverted phase contrast microscope, the passage 2 neurospheres (diameter of 80–90 μm) were individually transferred into nonadherent 96-well plates (one neurosphere/well) in 200 μL of medium using a sterile capillary tube. Then, the neurospheres were randomly divided into OGD + vehicle (DMSO), OGD + PFT-α (1 μM), OGD + PFT-α (5 μM) and OGD + PFT-a (10 μM) groups (n = 6 for each group). One hour later, OGD exposed neurospheres were transferred to normal growth conditions for 7 days. The diameter of the neurospheres was measured using a DP71 camera and Image-Pro Plus version 5.0.1 software^59^. The diameters for each treatment group were obtained from three independent experiments.

### Quantitative Real-time PCR

Total RNA was extracted using TRIzol reagent (Invitrogen). The cDNAs were synthesized as PrimeScript RT reagents Kit (TaKaRa) manufacturer’s instructions. Quantitative real-time polymerase chain reaction (qRT-PCR) was performed in triplicate and normalized to β-actin as endogenous control. Endogenous mRNA levels of p21, PUMA and β-actin were determined with SYBR PrimeScript RT-PCR Kit (TaKaRa). The PCR primers designed and synthesized by Sangon Biotech (Shanghai). P21Forward: GAGCAGTGCCCGAGTTAAGG, Reverse: TGGAACAGGTCGGACATCA. β-actin Fprward: CAACCTTCTTGCAGCTCCTC, Reverse: TTCTGACCCATACCCACCAT. PUMA Forward: CGTGTGGAGGAGGAGGAGT, Reverse: TAGTTGGGCTCCATTTCTGG. The relative quantitation value for each target gene was expressed as 2^−ΔΔCt^ as previous described^60^

### siRNA transfection *in vitro*

siRNA-p53 was from Santa Cruz Biotechnology. It was transfected into NSCs by liposome. The cells were incubated for 24 h, the culture media was refreshed and the cells were cultured for a further 24 h. The silencing effect of protein expression was confirmed by Western blot analysis.

### Statistics

Data were expressed as mean ± SEM. Comparisons among multiple groups were made with one-way ANOVA (one factor) or two-way ANOVA (two factors) followed by Bonferroni’s *post hoc* analysis. Comparisons between two groups were made using a two-tailed Student’s t-test. Data analysis was performed using SPSS version 13.0 (SPSS, Chicago, IL, USA). P < 0.05 was considered statistically significant.

## Additional Information

**How to cite this article**: Zhang, P. *et al*. Regenerative repair of Pifithrin-α in cerebral ischemia via VEGF dependent manner. *Sci. Rep*. **6**, 26295; doi: 10.1038/srep26295 (2016).
